# The Impact of Intravenous Lidocaine on ICP in Neurological Illness: A Systematic Review

**DOI:** 10.1155/2015/485802

**Published:** 2015-09-10

**Authors:** F. A. Zeiler, N. Sader, C. J. Kazina

**Affiliations:** ^1^Section of Neurosurgery, Department of Surgery, University of Manitoba, GB1-820 Sherbrook Street, Winnipeg, MB, Canada R3A 1R9; ^2^University of Manitoba, GB1-820 Sherbrook Street, Winnipeg, MB, Canada R3A 1R9

## Abstract

*Background*. The goal of our study was to perform a systematic review of the literature to determine the effect that intravenous (IV) lidocaine had on ICP in patients with neurological illness. *Methods*. All articles are from MEDLINE, BIOSIS, EMBASE, Global Health, Scopus, Cochrane Library, the International Clinical Trials Registry Platform (inception to March 2015). The strength of evidence was adjudicated using both the Oxford and GRADE methodology. *Results*. Ten original articles were considered for the final review. There were 189 patients studied. Seven studies focused on prophylactic pretreatment with IV lidocaine to determine if there would be an attenuation of ICP spikes during stimulation, with 4 displaying an attenuation of ICP. Three studies focused on a therapeutic administration of IV lidocaine in order to determine ICP reduction effects. All therapeutic studies displayed a reduction in ICP. *Conclusions*. We cannot make a strong definitive recommendation on the effectiveness of IV lidocaine on the attenuation of ICP spikes during stimulation. There currently exists both Oxford 2b and GRADE B literature to support and refute the attenuation of ICP spikes with IV lidocaine during stimulation. There currently exists Oxford 2b, GRADE B evidence to support ICP reduction with lidocaine when used as a therapeutic agent.

## 1. Introduction

Numerous medical therapies have been utilized in the treatment of elevated intracranial pressure (ICP) [1]. The majority of ICP therapies are directed at reducing cerebral blood volume, attenuation of edema, cerebrospinal fluid (CSF) diversion, and decreasing metabolic demands of the neural tissue, with the goal of maintaining adequate cerebral blood flow (CBF) and cellular preservation.

Specific concerns of ICP elevations occur during the peri-intubation period, due to the significant stimulation that occurs during definitive airway management. Lidocaine use, both intravenous (IV) and laryngotracheal (LT), has been reported to blunt the ICP elevations during intubation [2]. Though one would assume that the ICP mediated effects of lidocaine stem from its local anesthetic effect, there are other proposed mechanisms of ICP reduction via the IV route. Lidocaine injected IV has been shown in models to induce cerebral vasoconstriction leading to a decrease in cerebral blood volume and thus ICP [2, 3]. Furthermore, IV lidocaine leads to sodium channel inhibition and thus a reduction in cerebral activity and metabolic demands [3, 4], as well as excitotoxicity [5], leading to a potential ICP reduction effect.

To date few studies have documented the ICP effect of IV lidocaine in humans with neurological illness [6–16]. The goal of our study is to perform a systematic review of the literature on the use of intravenous lidocaine and its effects on ICP in patients with neurological illness.

## 2. Methods

A systematic review using the methodology outlined in the Cochrane Handbook for Systematic Reviewers [17] was conducted. The data was reported following the Preferred Reporting Items for Systematic Reviews and Meta-Analyses (PRISMA) [18]. The review question and search strategy were decided upon by the primary author (F. A. Zeiler) and supervisor (C. J. Kazina).

### 2.1. Search Question, Population, and Inclusion and Exclusion Criteria

The question posed for systematic review was the following: What is the effect of IV lidocaine on ICP in patients with neurological illness? We posed this broad question so as to include all eligible studies with objective ICP documentation during IV lidocaine administration, since we suspected the literature to be scarce.

All studies, prospective and retrospective, were included. Studies of any language were included, with relevant manuscripts translated in order to ensure that they met the predefined inclusion/exclusion criteria. The reason for an all-inclusive search was based on the small number of studies of any type identified by the primary author during a preliminary search of MEDLINE.

The primary outcome measure was documented effect of IV lidocaine on ICP. Secondary outcome measures were patient outcomes and adverse effects of lidocaine therapy.

Inclusion criteria were all studies including human subjects with neurological illness, prospective or retrospective studies of any size, any age category, any language, the use of IV lidocaine, and documentation of ICP response. Exclusion criteria were animal studies, those studies not documenting the ICP response, and the use of LT lidocaine only.

### 2.2. Search Strategy

MEDLINE, BIOSIS, EMBASE, Global Health, SCOPUS, and Cochrane Library from inception to March 2015 were searched using individualized search strategies for each database. The search strategy for MEDLINE can be seen in Appendix A of the supplementary material (see Supplementary Material available online at http://dx.doi.org/10.1155/2015/485802), with a similar search strategy utilized for the other databases. In addition, the World Health Organization's International Clinical Trials Registry Platform and https://clinicaltrials.gov/ were searched looking for studies planned or underway.

In addition, meeting proceedings for the last 5 years looking for ongoing and unpublished work based on lidocaine therapy in neurological patients were examined by hand searching the published proceedings for individual meetings. Both electronic and paper publications were searched when available. The meeting proceedings of the following professional societies were searched: Canadian Neurological Sciences Federation (CNSF), American Association of Neurological Surgeons (AANS), Congress of Neurological Surgeons (CNS), European Neurosurgical Society (ENSS), World Federation of Neurological Surgeons (WFNS), American Neurology Association (ANA), American Academy of Neurology (AAN), European Federation of Neurological Science (EFNS), World Congress of Neurology (WCN), Society of Critical Care Medicine (SCCM), Neurocritical Care Society (NCS), World Federation of Societies of Intensive and Critical Care Medicine (WFSICCM), American Society for Anesthesiologists (ASA), World Federation of Societies of Anesthesiologist (WFSA), Australian Society of Anesthesiologists, International Anesthesia Research Society (IARS), Society of Neurosurgical Anesthesiology and Critical Care (SNACC), Society for Neuroscience in Anesthesiology and Critical Care, and the Japanese Society of Neuroanesthesia and Critical Care (JSNCC).

Finally, reference lists of any review articles or systematic reviews on IV lidocaine, or ICP control, in neurologically ill patients were reviewed for relevant studies.

### 2.3. Study Selection

Utilizing two reviewers (F. A. Zeiler and N. Sader), a two-step review of all articles returned by our search strategies was performed. First, the reviewers independently screened all titles and abstracts of the returned articles to decide if they met the inclusion criteria. Second, full text of the chosen articles was then assessed to confirm if they met the inclusion criteria and that the primary outcome of ICP response was reported in the study. Any discrepancies between the two reviewers were resolved by discussion and a third party (C. J. Kazina) if necessary.

### 2.4. Data Collection

Data was extracted from the selected articles and stored in an electronic database. Data fields included patient demographics, type of study (prospective or retrospective), number of patients, dose of lidocaine, duration of therapy, effect on ICP, adverse effects, and patient outcome.

### 2.5. Quality of Evidence Assessment

Assessment of the level of evidence for each included study was conducted by two independent reviewers (F. A. Zeiler and C. J. Kazina), utilizing the Oxford criteria [19] and the Grading of Recommendation Assessment Development and Education (GRADE) criteria [20–25] for level of evidence. We elected on utilizing two different systems to grade level of evidence given the fact that these two systems are amongst the most commonly used systems. We believe this would allow a larger audience to follow our systematic approach in the setting of unfamiliarity with a particular grading system.

The Oxford criteria consist of a 5-level grading system for literature. Level 1 is split into subcategories 1a, 1b, and 1c which represent a systematic review of randomized control trials (RCT) with homogeneity, individual RCT with narrow confidence interval, and all or none studies, respectively. Oxford level 2 is split into 2a, 2b, and 2c representing systematic review of cohort studies with homogeneity of data, individual cohort study or low quality RCT, and outcomes research, respectively. Oxford level 3 is split into 3a and 3b representing systematic review of case-control studies with homogeneity of data and individual case-control study, respectively. Oxford level 4 represents case-series and poor cohort studies. Finally, Oxford level 5 represents expert opinion.

The GRADE level of evidence is split into 4 levels: A, B, C, and D. GRADE level A represents high evidence with multiple high quality studies having consistent results. GRADE level B represents moderate evidence with one high quality study, or multiple low quality studies. GRADE level C evidence represents low evidence with one or more studies with severe limitations. Finally, GRADE level D represents very low evidence based on either expert opinion or few studies with severe limitations.

Any discrepancies between the grading of the two reviewers were resolved via discussion and a third reviewer when required.

### 2.6. Statistical Analysis

A meta-analysis was not performed in this study due to the heterogeneity of data and study design within the articles identified.

## 3. Results

**Figure 1:**
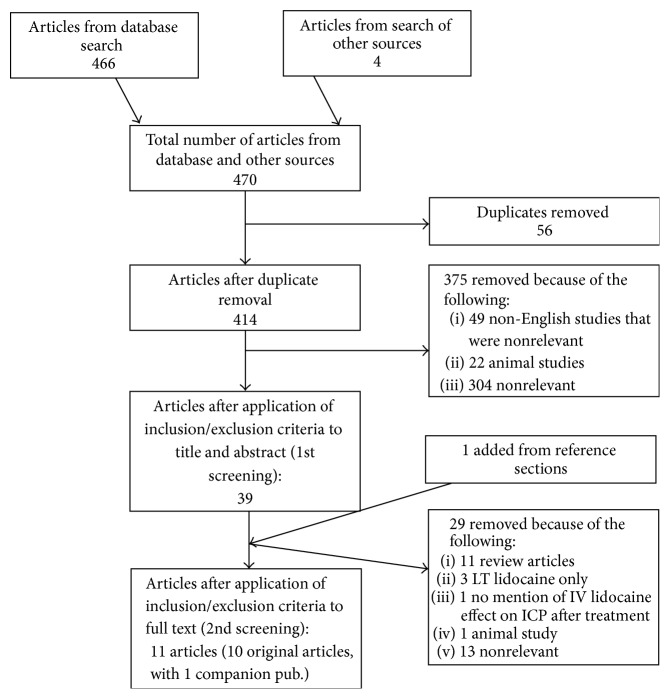
Flow diagram of search results.

The results of the search strategy across all databases and other sources are summarized in Figure 1. Overall a total of 470 articles were identified, with 466 from the database search and 4 from the search of published meeting proceedings. A total of 56 were removed due to duplication of reference, leaving 414 to review. By applying the inclusion/exclusion criteria to the title and abstract of these articles, we identified 39 articles that fit these criteria. An additional 1 article was added from reference sections of pertinent review articles, leaving a total of 40 full manuscripts to review. Of the 40 identified, 36 were from the database search and 4 were from published meeting proceedings. Applying the inclusion/exclusion criteria to the full text documents, only 11 articles were eligible for inclusion in the systematic review, with 8 from database and 3 from meeting proceeding sources. The 29 articles that were excluded were done so because either they did not report details around IV lidocaine administration, there was no documentation of ICP, they were review articles, or they were nonrelevant basic science articles.

Of the 11 articles identified, one article was a companion abstract publication [9] to a formal manuscript publication [8]. The data from this companion abstract [9] was included in the tables for completeness only and was not included in the data synthesis in order to prevent duplication of patient data. Thus, 10 original articles were included in the data review and data analysis. Two of the manuscripts were non-English papers requiring translation [13, 14], with one being French [14] and the other Italian [13].

Of the 10 original articles included in the review [6–8, 10–16], all were prospective studies. There were 4 prospective RCT [7, 10, 11, 14], 2 prospective nonrandomized trials [6, 12], and 4 prospective cohort studies [8, 13, 15, 16].

Four of the articles [8, 13, 15, 16] focused on achieving the ICP response to IV lidocaine in severe traumatic brain injury (TBI) patient populations, while 3 articles described malignant elective brain tumor patients [6, 7, 11], and 1 study mentioned hydrocephalus patients with elevated ICP^10^. Two studies failed to detail the patient populations and stated they were “post-operative neurosurgical patients” or “elective operative neurosurgical patients” and also failed to indicate if the patients had elevated ICP prior to lidocaine administration [12, 14]. The ICP was recorded via cranial monitoring in all but 1 study, which utilized lumbar cisternal pressure [14].

Overall, there were 189 patients across the 10 studies included in the review, with 133 given IV lidocaine and 56 serving as controls. The control patients were administered the following: esmolol (11), LT lidocaine (11), thiopental (10), placebo (10), steroid/glycerin (7), and steroids/glycerin/nitroglycerin (7).

There were 54 severe TBI patients, 62 elective tumor patients, and 30 hydrocephalus patients undergoing ventriculoperitoneal shunt (VPS) placement, and 43 patients were listed as “post-operative neurosurgical patients” [12] or “elective operative neurosurgical patients” [14].

The average age across all studies was 34.6 to 55 years. Five studies failed to document patient age [8, 10–12, 15]. Study demographics and patient characteristics can be seen in Table 1, while treatment characteristics and effect on ICP and adverse events are reported in Table 2.

### 3.1. IV Lidocaine Treatment Characteristics

Within the 10 studies identified [6–8, 10–16], 4 were prospective RCT [7, 10, 11, 14]. The first study [7] compared IV lidocaine (*n* = 10) versus saline placebo (*n* = 10) pretreatment, in elective tumor patients undergoing surgery, to determine the effect on ICP control during laryngoscopy. The dose of lidocaine used was a single 1.5 mg/kg IV bolus. The second study [10] compared 3 different IV lidocaine doses, in patients with hydrocephalus and elevated ICP, in order to determine the effect on ICP once the patient was under a general anesthetic. The doses of lidocaine studied were 1 mg/kg IV (*n* = 10), 1.5 mg/kg IV (*n* = 10), and 2 mg/kg IV (*n* = 10). The third study [11] compared IV lidocaine (*n* = 11) to LT lidocaine (*n* = 11), in elective tumor patients, in order to determine the effect on ICP during laryngoscopy. The doses of lidocaine used were 1.5 mg/kg IV bolus and 4 mL of 4% lidocaine instilled directly. The fourth study [14] compared pretreatment with IV lidocaine (*n* = 10) versus IV esmolol (*n* = 10), in elective operative neurosurgical patients, in order to determine the effect on ICP (measured via lumbar drain) during laryngoscopy. The doses of the medication used were lidocaine 1.5 mg/kg IV single dose and esmolol 1.5 mg/kg IV single dose.

There were 2 prospective nonrandomized studies [6, 12]. The first study [6] compared IV lidocaine (*n* = 10) versus thiopental (*n* = 10), in elective tumor operations with sustained ICP elevations after induction, in order to determine the impact on ICP control. The doses of medication studied were lidocaine 1.5 mg/kg IV single dose and thiopental 3 mg/kg IV single dose. The second study [12] compared steroids/glycerin/IV lidocaine (*n* = 7) versus steroids/glycerin (*n* = 7) and steroids/glycerin/nitroglycerin (*n* = 7), in postoperative neurosurgical patients, in order to determine the impact on ICP control. All patients received dexamethasone 5–10 mg three times daily and glycerin 200 gm/day. The doses of lidocaine and nitroglycerin were 1.5–3 mg/kg/min IV continuous infusion and 3–7 mcg/kg/min continuous infusion, respectively. The duration of the continuous infusions was not specified.

Four prospective cohort studies [8, 13, 15, 16] evaluated the effects of IV lidocaine on ICP control. The first study [8] prospectively followed 10 severe TBI patients with refractory ICP issues. This study documented the effects of pretreatment with 1.5 mg/kg single bolus IV lidocaine and saline boluses in every patient, in order to determine the impact on ICP during suctioning. The second study [13] prospectively followed 20 severe TBI patients with refractory ICP and compared the effect of no lidocaine, IV lidocaine, and LT lidocaine before treatment on ICP during suctioning in all patients. The doses of lidocaine used were 1.5 mg/kg IV single dose and 2 mL of 5% lidocaine instilled directly via the endotracheal tube. The third study [15] prospectively followed 15 severe TBI patients with refractory ICP and compared the effects of a variety of pretreatments (saline, fentanyl, thiopental, IV lidocaine/succinylcholine, and LT lidocaine) on ICP control during suctioning. The dose of lidocaine used was 1.5 mg/kg IV single bolus and LT 1.5 mg/kg instilled directly. The final study [16] prospectively followed 9 severe TBI patients to determine the effects of pretreatment with IV and LT lidocaine impacted ICP control during suctioning. Both IV and LT were administered in all patients during separate periods. The dose of lidocaine used was 1.5 mg/kg IV single dose and 2 mL of 4% lidocaine single direct instillation. The lidocaine was administered at different intervals prior to suctioning: 1, 3, 5, 10, and 15 minutes.

Seven studies evaluated IV lidocaine in a prophylactic manner prior to stimulation [7, 8, 11, 13–16], in order to determine the drugs ability to attenuate ICP elevations. Three studies evaluated IV lidocaine in a therapeutic manner, in order to determine the potential ICP reduction effects of the drug [6, 10, 12]. The lidocaine treatment characteristics can be seen in Table 2.

### 3.2. ICP Response

#### 3.2.1. Pretreatment Regimens

Among the 4 RCT [7, 10, 11, 14] manuscripts identified, 3 studies focused on pretreatment with IV lidocaine prior to laryngoscopy or suctioning [7, 11, 14]. The first study [7], comparing lidocaine to placebo, displayed an attenuation of ICP elevation favoring IV lidocaine. The lidocaine group displayed a max mean ICP elevation of 6 mm Hg, versus 16 mm Hg in the placebo group. The second study [11] comparing IV versus LT lidocaine displayed a significant reduction in baseline ICP in the IV group and attenuation of ICP elevation during laryngoscopy in the IV group. The LT lidocaine group displayed no effect on ICP. The third study [14] compared IV lidocaine versus esmolol and failed to display a difference in either group on ICP control. Both lidocaine and esmolol pretreatment failed to attenuate ICP elevations with intubation.

Within the 4 prospective cohort studies identified [8, 13, 15, 16], all focused on pretreatment with IV lidocaine prior to endotracheal suctioning. Two displayed a suppression of ICP elevations with IV lidocaine [8, 16], while the other 2 failed to demonstrate an attenuation of this response with IV lidocaine [13, 15]. However, one study which failed to display an attenuation of ICP during suctioning, did show a baseline reduction in ICP by 4–6 mm Hg prior to stimulation [15].

#### 3.2.2. Therapeutic Regimens

One RCT study was identified which studied the therapeutic effect of IV lidocaine on ICP [10]. This study compared different doses of lidocaine in hydrocephalus patients and displayed a significant reduction in ICP from baseline in all groups studied. The study also displayed a dose-dependent ICP reduction effect with the lowest dose (1.0 mg/kg) and the highest dose (2 mg/kg) displaying a 17.5% and 37.5% reduction, respectively.

Two nonrandomized trials studied the therapeutic effect of ICP lidocaine on ICP [6, 12]. The first displayed a mean decrease in ICP of 15 mm Hg, with a mean time to nadir of 66 ± 10 seconds [6]. This was equivalent to the thiopental comparison group. The second trial studied IV lidocaine (with steroids and glycerine) versus steroids/glycerine and steroids/glycerin/nitroglycerin [12]. The lidocaine group displayed a mean ICP reduction of 8.9 mm Hg over 24 hours of continuous infusion. The nitroglycerine group displayed similar results.

### 3.3. Adverse Effects of Lidocaine Therapy

Two studies recorded adverse events related to IV lidocaine therapy [10, 14]. The first displayed a dose related decrease in systolic blood pressure, with this effect documented at a dose of 2 mg/kg [10]. The second study displayed a trend to significant reductions in cerebral perfusion pressure during lidocaine administration [14].

The remaining studies included in the review [6–8, 11–13, 15, 16] failed to document any events or clearly state that “there were no adverse events.”

### 3.4. Functional Outcome

Patient functional outcome was not reported in any study, as the focus of the manuscripts was to determine the ICP effects of lidocaine.

### 3.5. Level of Evidence

Based on two independent reviewers (F. A. Zeiler and C. J. Kazina), there were a total of 10 studies reviewed. The question “What is the effect of IV lidocaine on ICP in patients with neurological illness?” was evaluated based on the Oxford and GRADE criteria for level of evidence.

#### 3.5.1. Oxford Level of Evidence: Pretreatment Regimens

Four studies were Oxford level 2b [7, 8, 11, 16] evidence supporting an attenuation of ICP elevation with IV lidocaine pretreatment prior to stimulation. Three studies were Oxford level 2b [13–15] evidence against the attenuation of ICP elevation with IV lidocaine pretreatment.

#### 3.5.2. Oxford Level of Evidence: Therapeutic Regimens

Three studies were Oxford level 2b [6, 10, 12] evidence supporting a decrease in ICP when IV lidocaine is utilized in a therapeutic manner.

#### 3.5.3. GRADE Level of Evidence: Pretreatment Regimens

Two studies were GRADE B [7, 11] and 2 were GRADE C [8, 16] evidence supporting an attenuation of ICP elevation with IV lidocaine pretreatment prior to stimulation. One study was GRADE B [14] and two were GRADE C [13, 15] evidence. One study was GRADE level B [10] and 2 were GRADE level C [5, 7] evidence against the attenuation of ICP elevation with IV lidocaine pretreatment.

#### 3.5.4. GRADE Level of Evidence: Therapeutic Regimens

Two studies were GRADE B [6, 10] and 1 was GRADE C [12] evidence supporting a decrease in ICP when IV lidocaine is utilized in a therapeutic manner.

A summary of all levels of evidence for individual articles can be seen in Table 3.

## 4. Discussion

Some important aspects of IV lidocaine therapy in the neurological population have been emphasized during this review. First, there lacks convincing evidence to suggest that IV lidocaine, when utilized as a pretreatment for stimulation (laryngoscopy/suctioning), has any impact on the attenuation of ICP spikes. There exists evidence to both support and refute this. Second, there exists evidence, though limited, to suggest an ICP reduction effect of IV lidocaine when used as a therapeutic measure. This ICP reduction effect was displayed in both patients with and without elevated ICP. However, there lacks objective evidence to suggest that either a reduction in cerebral metabolism, or cerebral vasoconstriction, are mechanisms leading to this ICP reduction. Third, comments on the ICP reduction effect of IV lidocaine when utilized therapeutically can only be made for bolus dosing alone. Only 1 study was identified using continuous infusions of lidocaine. Fourth, hypotension seems common with IV lidocaine therapy when utilized in both a pretreatment and therapeutic fashion. This hypotensive effect seems dose dependent [10]. Finally, patient outcome secondary to IV lidocaine therapy cannot be made at this time.

Several important limitations exist to this study. First, there were a small number of studies, making the conclusions of this review difficult to generalize to all neurological patients. Furthermore, the 43 patients had unclear neuropathology identified within the studies thus making it hard to determine if there was a pathology specific benefit to lidocaine therapy. Third, the use of other ICP directed medical therapies during lidocaine administration makes interpreting the data on ICP response difficult. The majority of the studies did not detail the other therapies that were being administered. Fourth, given the absence of outcome data reported, we cannot make any comments on the impact of IV lidocaine on functional outcome. Fifth, the degree of therapies applied to the TBI, hydrocephalus, and brain tumor patient populations were likely dramatically different. A full outline of ICP therapies was not present in the majority of studies, and this suspected variation between separate pathologies limits our ability to generalize the results for all patients with neurological illness. Finally, there is likely a significant publication bias with only those studies with positive results making it to publication within the literature.

Through this review, the ICP response to IV lidocaine has been thoroughly outlined. The current literature does not strongly support a “protective effect” of IV lidocaine during laryngoscopy/suctioning. The potential ICP reduction effect of IV lidocaine when used as a therapeutic measure seems to exist, though the exact mechanism is unclear. Further prospective trials need to be conducted to confirm the efficacy of IV lidocaine in both the pretreatment and therapeutic settings.

Future study should focus on the severe TBI population with lidocaine use both as a bolus dose based ICP reduction agent and in the context of a continuous infusion. The potential benefit of bolus based regimens could be assessed via comparing IV lidocaine bolus to either sedation bolus or hypertonic/hyperosmotic solutions bolus. Similarly, the continuous infusion dosing regimen for ICP reduction could be assessed in a prospective randomized fashion with implementation during crisis. One could also foresee the potential for early implementation of continuous IV lidocaine as a prophylactic measure for ICP reduction, in an attempt to reduce the sedation and hypertonic/hyperosmotic agent requirements. 

*Final Recommendations*. Overall, we cannot make a strong definitive recommendation on the effectiveness of pretreatment IV lidocaine for the attenuation of ICP elevations during stimulation. There is Oxford 2b, GRADE B level of evidence supporting both the attenuation of ICP increases, and no impact on ICP control, with pretreatment IV lidocaine.

Therapeutic use of IV lidocaine for ICP reduction currently displays Oxford 2b, GRADE B evidence supporting ICP reduction with lidocaine administration.

## 5. Conclusions

There lacks strong literature to support the effectiveness of pretreatment IV lidocaine for the attenuation of ICP elevations during stimulation. There is Oxford 2b, GRADE B level of evidence supporting both the attenuation of ICP increases, and no impact on ICP control, with pretreatment IV lidocaine. Therapeutic use of IV lidocaine for ICP reduction currently displays Oxford 2b, GRADE B evidence supporting ICP reduction with lidocaine administration.

## Supplementary Material

Appendix A of the supplementary materials contains the search strategy utilized for the Medline search. A similar search strategy was used for the other databases described within the manuscript.

## Figures and Tables

**Table 1:** Study characteristics and patient demographics.

Reference	Number of patients	Study type	Article location	Mean age (years)	Patient characteristics	Primary and secondary goal of study
Bedford et al. [6]	20 *Lidocaine* (*n* = 10) *Thiopental* (*n* = 10)	Prospective nonrandomized	Manuscript	*Lidocaine* = 51.1 ± 4.4 *Thiopental* = 47.5 ± 5.0	Brain tumors undergoing elective resection having sustained ICP elevations for >30 sec after induction *Anesthetic*: thiopental, nitrous oxide/O_2_, and morphine	*Primary*: to determine the effect of IV lidocaine versus thiopental on ICP control for sustained ICP elevations *Secondary*: none stated

Bedford et al. [7]	20 *Lidocaine* (*n* = 10) *Placebo* (*n* = 10)	Prospective randomized control trial	Meeting abstract	*Lidocaine* = 49 ± 6 *Placebo* = 55 ± 6	Brain tumors undergoing elective resection *Anesthetic*: thiopental, nitrous oxide/O_2_, and morphine	*Primary*: to determine the effect of IV lidocaine versus saline (at induction) on ICP during laryngoscopy *Secondary*: none stated

Donegan and Bedford [8]	10	Prospective cohort	Manuscript	Unknown	Severe TBI with ICP > 20 mm Hg during suctioning *Concurrent ICP therapy*: hyperventilation (pCO_2_ goal 30 mm Hg), mannitol, dexamethasone, and pentobarbital (*n* = 5)	*Primary*: to determine the effect of IV lidocaine versus saline on ICP during suctioning *Secondary*: not stated

^*∗*^Donegan et al. [9]	9	Prospective cohort	Meeting abstract	Unknown	Severe TBI with ICP > 20 mm Hg during suctioning *Concurrent ICP therapy*: hyperventilation (pCO_2_ goal 30 mm Hg), mannitol, dexamethasone, and pentobarbital (*n* = 5)	*Primary*: to determine the effect of IV lidocaine versus saline on ICP during suctioning *Secondary*: not stated

Grover et al. [10]	30	Prospective randomized trial	Manuscript	Age > 5 years	Clinically raised ICP and undergoing VPS surgery *Anesthetic*: thiopental induction with suxamethonium NMBA Maintenance with 66% nitrous oxide/oxygen mix, pancuronium, and morphine	*Primary*: to determine the effect of different IV lidocaine doses on ICP *Secondary*: not stated

Hamill et al. [11]	22 *LT Lidocaine* (*n* = 11) *IV Lidocaine* (*n* = 11)	Prospective randomized trial	Manuscript	Unknown	Brain tumors undergoing elective resection *Anesthetic*: thiopental, succinylcholine, and 50% nitrous oxide/oxygen mix	*Primary*: to determine effect of LT versus IV lidocaine on ICP during laryngoscopy *Secondary*: HR and MABP

Hirayama et al. [12]	21 patients *Steroid/glycerin* (*n* = 7) *Steroid/glycerin/lidocaine* (*n* = 7) *Steroid/glycerin/nitroglycerin* (*n* = 7)	Prospective nonrandomized	Meeting abstract	Unknown	“Postoperative” neurosurgery patients *Concurrent ICP therapy*: dexamethasone 5–10 mg TID, glycerin 200 gm/day	*Primary*: to determine the effects of IV lidocaine versus nitroglycerin on ICP *Secondary*: not stated

Montarry et al. [13]	20	Prospective cohort	Manuscript	32	Severe TBI *Concurrent ICP therapy*: thiopental, maintained negative fluid balance, pCO_2_ goal 28 mm Hg	*Primary*: to determine the effects of IV versus LT lidocaine on ICP control during endotracheal suctioning *Secondary*: cerebral perfusion pressure

Samaha et al. [14]	22 *Esmolol* (*n* = 11) *Lidocaine* (*n* = 11)	Prospective randomized trial	Manuscript	*Esmolol*: 50 ± 13 *Lidocaine*: 47 ± 16	Elective neurosurgical patients (tumors and aneurysm clippings) ^*∗*^ *ICP measured via LD Anesthetic*: thiopental induction, sevoflurane, maintenance, pCO_2_ goal 33–35 mm Hg	*Primary*: to determine the effect of esmolol versus lidocaine IV bolus (130 sec before intubation) on lumbar cistern pressures during laryngoscopy (ICP measured before, during, and 2 and 5 min after intubation) *Secondary*: MABP, CPP

White et al. [15]	15	Prospective cohort	Manuscript	Unknown	Severe TBI with elevations of ICP > 20 mm Hg during or immediately after suctioning *Concurrent ICP therapy*: sedation with diazepam and morphine, hyperventilation (pCO_2_ 25–30 mm Hg)	*Primary*: to determine the effects of different pretreatment (saline, fentanyl, thiopental, lidocaine, and succinylcholine) on ICP during suctioning *Secondary*: MABP

Yano et al. [16]	9	Prospective cohort	Manuscript	34.6 (range: 16 to 71)	Severe TBI *Concurrent ICP therapy*: not specific, hyperventilated to pCO_2_ goal of 25 to 30 mm Hg	*Primary*: to determine the effect of IV and LT lidocaine on ICP during suctioning *Secondary*: not stated

*n* = number of patients, HR = heart rate, MABP = mean arterial blood pressure, ICP = intracranial pressure, CPP = cerebral perfusion pressure, CSF = cerebrospinal fluid, mm** **Hg = millimeters of mercury, IV = intravenous, LT = laryngotracheal, TBI = traumatic brain injury, LD = lumbar drain, and sec = second. ^*∗*^Donegan and Bedford [8] and Donegan et al. [9] are companion publications, with Donegan et al. [9] representing the meeting abstract published prior to the full manuscript [8]. The data from Donegan et al. [9] is not included in the synthesis of data and is only included in the tables for completeness.

**Table 2:** Lidocaine treatment characteristics and ICP response.

Reference	Lidocaine dose	Mean duration of lidocaine administration (days)	ICP response	Other primary/secondary outcomes	Adverse effects to lidocaine	Conclusions
Bedford et al. [6]	1.5 mg/kg IV bolus x1 (*n* = 10) *Thiopental dose* (*n* = 10) = 3 mg/kg IV x1	Single bolus dose	*Lidocaine*: decrease in mean ICP from 28 mm Hg to 13 mm Hg, with nadir reached at a mean 66 ± 10 sec *Thiopental*: decrease in mean ICP from 33 mm Hg to 14 mm Hg, with a nadir reached at a mean 48 ± 9 sec	Mean MABP decrease of 26 mm Hg with thiopental No MABP changes with lidocaine	None described	Lidocaine and thiopental are equally an effect in reduction of ICP via IV bolus, with lidocaine preserving systemic hemodynamics

Bedford et al. [7]	1.5 mg/kg IV bolus x1 (*n* = 10) *Saline* (*n* = 10) = *same volume*	Single bolus dose	Pretreatment with IV lidocaine led to a maximum mean ICP increase of 6 mm Hg and 4 mm Hg at 30 and 60 sec Saline placebo mean increase in ICP of 16 mm Hg and 13 mm Hg at 30 and 60 sec	MABP increase was less with lidocaine compared to placebo	None described	IV lidocaine during laryngoscopy leads to an attenuation of ICP elevation compared to saline.

Donegan and Bedford [8]	1.5 mg/kg IV bolus x1 *Saline was given in the same volume as lidocaine Patients received both therapies*	Single bolus dose	Lidocaine presuction led to a mean increase in ICP of 3.4 ± 6.2 mm Hg and 1.8 ± 2.6 mm Hg in those on and off barbiturates during suctioning Saline placebo presuction led to a mean increase in ICP 19 ± 4.7 mm Hg and 5.7 ± 3.2 mm Hg in those on and off barbiturates during suctioning	No significant difference in MABP changes during suctioning	None described	Lidocaine presuctioning leads to an attenuation of ICP elevations compared to saline

^*∗*^Donegan et al. [9]	1.5 mg/kg IV bolus x1 *Saline was given in the same volume as lidocaine Patients received both therapies*	Single bolus dose	Lidocaine pretreatment led to a significant attenuation in ICP elevation with suctioning	Not stated	None described	Lidocaine presuctioning leads to an attenuation of ICP elevations compared to saline

Grover et al. [10]	Three groups: Group 1 (*n* = 10): 1 mg/kg IV bolus x1 Group 2 (*n* = 10): 1.5 mg/kg bolus x1 Group 3 (*n* = 10): 2 mg/kg bolus x1	Single bolus dose	All groups displayed a significant decrease in ICP within 2 min of lidocaine administration Group 3 displayed a 37.5% reduction in mean ICP, and Group 1 displayed a 17.5% mean reduction	Group 3 was the only group to display significant drop in SBP	SBP decreased with 2 mg/kg IV bolus dose	IV lidocaine bolus leads to significant reductions in ICP High lidocaine dosing may lead to drops in SBP

Hamill et al. [11]	LT (*n* = 11): 4 mL 4% lidocaine endotracheal IV (*n* = 11): 1.5 mg/kg IV bolus x1	Single dose	IV lidocaine led to a significant decrease in baseline ICP with no elevations during laryngoscopy LT lidocaine failed to decrease ICP and all patients had a significant elevation in ICP during intubation	Significant HR and MABP increase in LT group	None described	IV lidocaine led to decrease in baseline ICP and attenuated ICP elevations during laryngoscopy

Hirayama et al. [12]	All had dexamethasone and glycerin, with *n* = 7 this therapy in isolation. Lidocaine (*n* = 7): 10% lidocaine continuous infusion at 1.5–3 mg/min Nitroglycerine (*n* = 7): 3–7 mcg/kg/min	Continuous infusion	The addition of lidocaine to glycerin therapy led to a reduction in ICP spikes, with a mean reduction in ICP of 8.9 mm Hg over 24 hours The addition of nitroglycerin to glycerin led to a mean reduction of ICP of 7.4 mm Hg over 24 hours	Not stated	None described	Both IV lidocaine and nitroglycerin in the presence of glycerin therapy lead to ICP reductions at 24 hours

Montarry et al. [13]	All patients underwent suctioning without lidocaine, then with IV, and finally with LT *Lidocaine IV: 1.5 mg/kg of 2% lidocaine bolus x1 Lidocaine LT: 5% lidocaine*	Single dose	No difference in the ICP over 6 min for no lidocaine, or either of the lidocaine routes, during suctioning. 10 patients had documented ICP > 20 mm Hg and had the same lidocaine treatment *Lidocaine IV led to a mild reduction in ICP over 6 min by approx. 2 mm Hg LT lidocaine did not have any significant effect compared to baseline*	No difference in CPP	Not described	Lidocaine IV or LT did not lead to a suppression of ICP during suctioning Lidocaine IV bolus during acute ICP elevations may lead to mild reduction in ICP.

Samaha et al. [14]	*Lidocaine*: 1.5 mg/kg IV x1 *Esmolol*: 1.5 mg/kg IV x1	Single dose	Postintubation ICP rose significantly in both groups *Esmolol*: increased from 11 ± 6 mm Hg to 17 ± 10 mm Hg *Lidocaine*: increased from 10 ± 6 mm Hg to 16 ± 9 mm Hg	Significant decrease in CPP in both groups during intubation *Esmolol decreased from a mean of 92 to 62 mm Hg Lidocaine decreased from a mean of 96 to 68 mm Hg * *After intubation* *CPP increased to 99 ± 23 mm Hg and 99 ± 17 mm Hg in the esmolol and lidocaine groups*	Not described	Both lidocaine and esmolol failed to attenuate the elevation in CPP and ICP after intubation

White et al. [15]	Every patient received all treatments during individual suctioning episodes Saline = 2 mL Fentanyl = 1 mcg/kg Thiopental = 3 mg/kg Lidocaine = 1.5 mg/kg + succinylcholine = 1.5 mg/kg Lidocaine LT alone = 1.5 mg/kg	Single bolus dose	Lidocaine/succinylcholine IV and thiopental lead to a mean decrease in ICP by 4–6 mm Hg but had no effect on the ICP during suctioning LT lidocaine had more effect at attenuating cough and ICP elevations during suctioning. However, during instillation it initiated coughing and led to ICP spikes Fentanyl had no effect on ICP	MABP not affected by any regimen	None described	Lidocaine IV leads to ICP reduction but not attenuation of cough mediated ICP spikes LT lidocaine may be superior in preventing coughing related ICP spikes

Yano et al. [16]	1.5 mg/kg IV bolus dose at the following intervals prior to suctioning: 1, 3, 5, 10, and 15 min LT lidocaine = 2 mL of 4% lidocaine across the same intervals	Single bolus doses	Neither IV of LT lidocaine lowered baseline ICP, but both suppressed ICP elevations with suctioning LT lidocaine led to a lower peak ICP compared to IV	Not stated	None described	Both IV and LT lidocaine suppress ICP elevations during suctioning

*N* = number of patients, mg = milligram, mcg = microgram, mL = milliliters, wt = weight, kg = kilogram, hr = hour, min = minute, HR = heart rate, MABP = mean arterial blood pressure, ICP = intracranial pressure, CPP = cerebral perfusion pressure, CSF = cerebrospinal fluid, mm Hg = millimeters of mercury, IV = intravenous, LT = laryngotracheal, TBI = traumatic brain injury, LD = lumbar drain, and sec = second. ^*∗*^Donegan and Bedford [8] and Donegan et al. [9] are companion publications, with Donegan et al. [9] representing the meeting abstract published prior to the full manuscript [8]. The data from Donegan et al. [9] is not included in the synthesis of data and is only included in the tables for completeness.

**Table 3:** Oxford and GRADE level of evidence.

Reference	Study type	Oxford level of evidence	GRADE level of evidence
Bedford et al. [6]	Prospective nonrandomized	2b	B
Bedford et al. [7]	Prospective randomized trial	2b	B
Donegan and Bedford [8]	Prospective cohort	2b	C
^*∗*^Donegan et al. [9]	Prospective cohort	2b	C
Grover et al. [10]	Prospective randomized trial	2b	B
Hamill et al. [11]	Prospective randomized trial	2b	B
Hirayama et al. [12]	Prospective nonrandomized	2b	C
Montarry et al. [13]	Prospective cohort	2b	C
Samaha et al. [14]	Prospective randomized trial	2b	B
White et al. [15]	Prospective cohort	2b	C
Yano et al. [16]	Prospective cohort	2b	C

^*∗*^Donegan and Bedford [8] and Donegan et al. [9] are companion publications, with Donegan et al. [9] representing the meeting abstract published prior to the full manuscript [8]. The data from Donegan et al. [9] is not included in the synthesis of data and is only included in the tables for completeness.
