# Supplementary material for: The Impact of Intravenous Lidocaine on ICP in Neurological Illness: A Systematic Review
Source: Crit Care Res Pract. 2015 Sep 10;2015:485802. doi: 10.1155/2015/485802 (PMC4581506; doi:10.1155/2015/485802)
Supplement: Supplementary file 1 — Appendix A of the supplementary materials contains the search strategy utilized for the Medline search. A similar search strategy was used for the other databases described within the manuscript. [file 485802.f1.pdf]

Appendix A: Medline Search Strategy

1 brain injury.mp. [mp=ti, ab, sh, hw, tn, ot, dm, mf, dv, kw, bt, id, cc, nm, kf, px, rx, an, ui]

2 brain illness.tw.

3 neurological injury.mp. [mp=ti, ab, sh, hw, tn, ot, dm, mf, dv, kw, bt, id, cc, nm, kf, px, rx, an, ui]

4 neurological injury.tw.

5 traumatic brain injury.mp. [mp=ti, ab, sh, hw, tn, ot, dm, mf, dv, kw, bt, id, cc, nm, kf, px, rx, an, ui]

6 traumatic brain injury.tw.

7 subarachnoid hemorrhage.mp. [mp=ti, ab, sh, hw, tn, ot, dm, mf, dv, kw, bt, id, cc, nm, kf, px, rx, an, ui]

8 subarachnoid hemorrhage.tw.

9 stroke.mp. [mp=ti, ab, sh, hw, tn, ot, dm, mf, dv, kw, bt, id, cc, nm, kf, px, rx, an, ui]

10 stroke.tw.

11 brain tumor.mp. [mp=ti, ab, sh, hw, tn, ot, dm, mf, dv, kw, bt, id, cc, nm, kf, px, rx, an, ui]

12 brain tumor.tw.

13 neurological illness.mp. [mp=ti, ab, sh, hw, tn, ot, dm, mf, dv, kw, bt, id, cc, nm, kf, px, rx, an, ui]

14 neurological illness.tw.

15 brain illness.mp. [mp=ti, ab, sh, hw, tn, ot, dm, mf, dv, kw, bt, id, cc, nm, kf, px, rx, an, ui]

16 brain illness.tw.

17 hydrocephalus.mp. [mp=ti, ab, sh, hw, tn, ot, dm, mf, dv, kw, bt, id, cc, nm, kf, px, rx, an, ui]

18 hydrocephalus.tw.

19 neurological pathology.mp. [mp=ti, ab, sh, hw, tn, ot, dm, mf, dv, kw, bt, id, cc, nm, kf, px, rx, an, ui]

20 neurological pathology.tw.

21 neurosurgical pathology.mp. [mp=ti, ab, sh, hw, tn, ot, dm, mf, dv, kw, bt, id, cc, nm, kf, px, rx, an, ui]

22 neurosurgical pathology.tw.

23 neuropathology.mp. [mp=ti, ab, sh, hw, tn, ot, dm, mf, dv, kw, bt, id, cc, nm, kf, px, rx, an, ui]

24 neuropathology.tw.

25 lidocaine.mp. [mp=ti, ab, sh, hw, tn, ot, dm, mf, dv, kw, bt, id, cc, nm, kf, px, rx, an, ui]

26 lidocaine.tw.

27 lidocaine hydrochloride.mp. [mp=ti, ab, sh, hw, tn, ot, dm, mf, dv, kw, bt, id, cc, nm, kf, px, rx, an, ui]

28 lidocaine hydrochloride.tw.

29 lignocaine.mp. [mp=ti, ab, sh, hw, tn, ot, dm, mf, dv, kw, bt, id, cc, nm, kf, px, rx, an, ui]

30 lignocaine.tw.

31 xylocaine.mp. [mp=ti, ab, sh, hw, tn, ot, dm, mf, dv, kw, bt, id, cc, nm, kf, px, rx, an, ui]

32 xylocaine.tw.

33 anestacon.mp. [mp=ti, ab, sh, hw, tn, ot, dm, mf, dv, kw, bt, id, cc, nm, kf, px, rx, an, ui]

34 anestacon.tw.

35 lidoderm.mp. [mp=ti, ab, sh, hw, tn, ot, dm, mf, dv, kw, bt, id, cc, nm, kf, px, rx, an, ui]

36 lidoderm.tw.

37 xycaine.mp. [mp=ti, ab, sh, hw, tn, ot, dm, mf, dv, kw, bt, id, cc, nm, kf, px, rx, an, ui]

38 xycaine.tw.

39 esracaine.mp. [mp=ti, ab, sh, hw, tn, ot, dm, mf, dv, kw, bt, id, cc, nm, kf, px, rx, an, ui]

40 esracaine.tw.

41 alphacaine.mp. [mp=ti, ab, sh, hw, tn, ot, dm, mf, dv, kw, bt, id, cc, nm, kf, px, rx, an, ui]

42 alphacaine.tw.

43 octocaine.mp. [mp=ti, ab, sh, hw, tn, ot, dm, mf, dv, kw, bt, id, cc, nm, kf, px, rx, an, ui]

44 octocaine.tw.

45 dalcaine.mp. [mp=ti, ab, sh, hw, tn, ot, dm, mf, dv, kw, bt, id, cc, nm, kf, px, rx, an, ui]

46 dalcaine.tw.

47 xylesthesin.mp. [mp=ti, ab, sh, hw, tn, ot, dm, mf, dv, kw, bt, id, cc, nm, kf, px, rx, an, ui]

48 xylesthesin.tw.

49 icp.mp. [mp=ti, ab, sh, hw, tn, ot, dm, mf, dv, kw, bt, id, cc, nm, kf, px, rx, an, ui]

50 icp.tw.

51 intracranial pressure.mp. [mp=ti, ab, sh, hw, tn, ot, dm, mf, dv, kw, bt, id, cc, nm, kf, px, rx, an, ui]

52 intracranial pressure.tw.

53 cranial pressure.mp. [mp=ti, ab, sh, hw, tn, ot, dm, mf, dv, kw, bt, id, cc, nm, kf, px, rx, an, ui]

54 cranial pressure.tw.

55 intracranial hypertension.mp. [mp=ti, ab, sh, hw, tn, ot, dm, mf, dv, kw, bt, id, cc, nm, kf, px, rx, an, ui]

56 intracranial hypertension.tw.

57 high icp.mp. [mp=ti, ab, sh, hw, tn, ot, dm, mf, dv, kw, bt, id, cc, nm, kf, px, rx, an, ui]

58 high icp.tw.

59 high pressure.mp. [mp=ti, ab, sh, hw, tn, ot, dm, mf, dv, kw, bt, id, cc, nm, kf, px, rx, an, ui]

60 high cranial pressure.tw.

61 high pressure.tw.

62 high cranial pressure.mp. [mp=ti, ab, sh, hw, tn, ot, dm, mf, dv, kw, bt, id, cc, nm, kf, px, rx, an, ui]

63 cerebrospinal fluid pressure.mp. [mp=ti, ab, sh, hw, tn, ot, dm, mf, dv, kw, bt, id, cc, nm, kf, px, rx, an, ui]

64 cerebrospinal fluid pressure.tw.

65 spinal fluid pressure.mp. [mp=ti, ab, sh, hw, tn, ot, dm, mf, dv, kw, bt, id, cc, nm, kf, px, rx, an, ui]

66 spinal fluid pressure.tw.

67 1 or 2 or 3 or 4 or 5 or 6 or 7 or 8 or 9 or 10 or 11 or 12 or 13 or 14 or 15 or 16 or 17 or 18 or 19 or 20 or 21 or 22 or 23 or 24

68 25 or 26 or 27 or 28 or 29 or 30 or 31 or 32 or 33 or 34 or 35 or 36 or 37 or 38 or 39 or 40 or 41 or 42 or 43 or 44 or 45 or 46 or 47 or 48

69 49 or 50 or 51 or 52 or 53 or 54 or 55 or 56 or 57 or 58 or 59 or 60 or 61

70 67 and 68

71 69 and 70

72 remove duplicates from 71
